# In Vitro Evaluation of Tc-99m Radiopharmaceuticals for Gastric Emptying Studies

**DOI:** 10.4274/Mirt.75047

**Published:** 2014-02-05

**Authors:** Türkan Ertay, Amet Semih Doğan, Özden Ülker, Hatice Durak

**Affiliations:** 1 Dokuz Eylül University, Department of Nuclear Medicine, İzmir, Turkey

**Keywords:** Gastric emptying, scintigraphy, radiopharmaceutical, technetium, Tc-99m sulfur colloid, nanoparticles, Tc-99m-MAA, Tc 99m-DTPA

## Abstract

**Objective**: Gastrointestinal motility and functional motility disorders causing either delayed or accelerated gastric emptying (GE) may result in similar symptoms including nausea, vomiting, early satiety, fullness, bloating, and abdominal discomfort or pain. Hence, it is important to evaluate patients for both rapid and delayed GE in the same test. The gold standard technique to measure GE is scintigraphy by radiolabeled test meals. The aim of this study was to test alternative Tc-99m agents to label eggs as the solid meal and compare to Tc-99m sulfur colloid (SC) for gastric emptying studies.

**Methods**: In search of alternative agents for gastric emptying studies, we mixed and fried eggs with four different particulate compounds (Tc-99m labeled SC, tin colloid, nanocolloid and MAA), as well as with free pertechnetate and Tc-99m DTPA. We then measured the stability of these compounds in simulated gastric juice.

**Results**: Our experiments demonstrated that in addition to Tc-99m sulfur colloid;Tc-99m MAA, Tc-99m nanocolloid and Tc-99m tin colloid also appear to make stable complexes with eggs in acidic environment.

**Conclusion**: Therefore, these agents may be used for gastric emptying studies which could be more practical in routine conditions.

**Conflict of interest:**None declared.

## Abstract (tr)

**Amaç**: Gastrointestinal motilite ve fonksiyonel motilite bozuklukları erken ya da geç mide boşalmasından kaynaklanan bulantı, kusma, erken doyma ve şişkinlik gibi abdominal rahatsızlık ya da ağrıya neden olan benzer semptomlar gösterir. Bu yüzden hastanın erken veya geç mide boşalma zamanını aynı test ile ölçmek önemlidir. Gastrik boşalmayı ölçmek için, altın standart yöntem, radyoişaretli besinler kullanılarak yapılan sintigrafi tekniğidir. Bu çalışmanın amacı alternatif Tc-99m radyofaramasötiklerini katı besin olarak yumurta ile birleştirip, mide boşalma sintigrafisi için kullanılabilirliğini Tc-99m sulfur kolloid (SC) ile karşılaştırarak test etmektir.

**Yöntem**: Çalışmada mide boşalma sintigrafisi için alternatif radyofarmasötikler olarak dört farklı partiküllü radyofarmasötik (Tc-99m ile işaretli SC, kalay kolloid, nano kolloid ve MAA) ile teknesyum perteknetat ve Tc-99m DTPA bileşikleri yumurta ile karıştırıldı ve pişirildi. Bu bileşiklerin stabiliteleri mide suyu benzeri ortamda değerlendirildi.

**Bulgular**: Sonuçlar Tc-99m SC’e ilaveten Tc-99m MAA, Tc-99m nano kolloid ve Tc-99m kalay kolloidin de asidik ortamda yumurta ile stabil kompleksler oluşturduğunu göstermiştir. Mide boşalma sintigrafisi için bu ajanların da kullanılabilmesi günlük çalışma koşullarında kolaylık sağlayacaktır.

## INTRODUCTION

Gastroparesis is a clinical syndrome characterized by delayed gastric emptying (GE) in the absence of mechanical gastric outlet obstruction. Symptoms may include nausea, vomiting, early satiety, abdominal pain, and bloating. It is a condition of abnormal gastric motility, which presents with symptoms suggesting delayed GE in the absence of an anatomic obstruction. The true prevalence of gastroparesis is unknown, but it may be present in as many as 65% of diabetic patients and 25%-40% of patients with upper gastrointestinal dyspepsia. Delayed GE may be idiopathic as in nonulcer dyspepsia (NUD) or it may accompany other disorders such as diabetes mellitus, gastroesophageal reflux disease (GERD), or vagotomy and gastric surgery for peptic ulcer disease (1,2,3). Delayed GE has been reported in 30-50% of patients with NUD (4) and in about 20-40% of patients with GERD (5,6,7). Gastric emptying (GE) studies are ordered to confirm or exclude the presence of gastroparesis as a cause of a patient’s symptoms (3). Gastric emptying scintigraphy (GES) is commonly performed to evaluate patients with symptoms that suggest an alteration of gastric emptying and/or motility (2).

The first use of radionuclides to measure GE was published in 1966 (2). Since then, it has become the standard for the measurement of gastric motility in clinical practice, because it provides a physiologic, noninvasive, and quantitative measurement of GE. GES is generally regarded as the gold standard to evaluate GE (8). As currently performed in most imaging centers, GES is limited to evaluation of a potential delay or acceleration in GE of a solid radiolabeled meal (3,4). A wide range of radiolabeled test meals have been used for gastric emptying scintigraphy. The aim of this study was to test alternative Tc-99m agents to label eggs as the solid meal and compare to Tc-99m sulfur colloid for gastric emptying studies.

## MATERIALS AND METHODS

As alternative agents for gastric emptying studies, we examined four different particulate compounds (Tc-99m labeled sulphur colloid (SC), nanocolloid, Tin colloid and macroaggregated albumin (MAA), as well as Tc-99m pertechnetate (TcO4-) and Tc-99m diethylenetriamin penta acetic acid (DTPA).

**Labeling Method**

Two whole eggs were transferred from their shells to a cup and then beaten for 1 min with a metal fork until the yolks and whites are blended. 18.5 MBq (0.5 mCi) Tc-99m SC was mixed with the eggs. The egg -Tc-99m SC mixture was poured in the pan and cooked to a firm consistency, turning the eggs to ensure complete cooking.

**Stability Test Method**

Each labeled cooked egg preparation was transferred to a plate and allowed to cool to room temperature and then were chopped to small pieces with a metal fork to simulate chewing. In order to simulate the acidic milieu of gastric juice, we added 1 N HCl in 20 ml saline and kept the pH between 2 and 3 and stirred. Labeled eggs were placed in centrifuge vials in acidic media. The vials were capped and placed in a water bath at 37°C for up to 90 minutes. The labeled solid food was homogenized in the simulated gastric juice, mixed every 15 minutes and centrifuged at 1000 rpm, 90 minutes after labeling. Aliquot was taken to determine proportion of radioactivity in the supernatant after centrifugation. The activity of the supernatant and the sediment was measured by gamma counting. Labeling efficiencies (LE) of the agents were calculated as: 

LE(%)= Sediment activity/Total activity x 100

The same procedure was applied for Tc-99m Tincolloid, Tc-99m Nanocolloid, Tc-99m MAA, Tc-99m DTPA and Tc-99m pertechnetate. The stability of the labeled solid food in simulated gastric juice was measured in three consecutive experiments. 

## RESULTS

The labeling efficiency (LE) of the particulate agents (Tc-99m SC, Tc-99m Tin Colloid, Tc-99m Nanocolloid and Tc-99m MAA) were between 90-100%. LE of Tc-99m pertechnetate and Tc-99m DTPA were between 60-80% at 90 minutes. The LE results are presented in the table (Table 1). 

## DISCUSSION

Historically, many attempts to achieve a radiolabeled solid meal did not validate the stability of the radiolabel and resulted in use of test meals in which the radiolabel dissociated from the solid particles (9). The first test meal with validated labeling was in vivo 99mTc-sulfur colloid chicken liver (4). Since then, many other meals have been used, but not all have been adequately tested for stability in gastric fluid (4,9). The meal should be ingested as quickly as possible, optimally within 10 minutes (4). Immediately after completion of the meal, the scintigraphic images are obtained via a gamma camera during a period of 90 minutes dynamically while the patient is in a supine or upright position (3,5,10).

The most widely used technique is labeling eggs with Tc-99m SC (11). However, there continues to be a lack of standardization of the test, which primarily relates to differences in meals and the duration of imaging.

There are also differences in the quantitative data reported, for example, half-time of emptying, rate of emptying (percent per minute), or the percent retention or emptying at different time points during the study (6).

The routine imaging time for the egg meals is 90 min. Delayed imaging is performed if the patient does not reach the half-time of emptying by the end of the routine imaging time (12).

For many years, gastric emptying tudies were performed using various local protocols and different radiolabeled meals (2,13,14,15).

A wide range of radiolabeled test meals have been used for gastric emptying scintigraphy. Knight et al. tested the stability of 99mTc-sulfur colloid labeled liquid egg white and whole egg and also evaluated the cooking methods by either microwaving or frying on a griddle. They concluded 99mTc-SC binds better to egg whites compared with whole eggs, they state that stability of a radiolabeled solid test meal for gastric emptying studies must be established both for its constituents and for the method of preparation (7).

Thomforde et al. measured transit scintigraphically after ingestion of an egg meal containing 99mTc-Amberlite pellets. They prospectively evaluated 35 patients referred to their laboratory with suspected fast or slow gastric emptying. They reported that selective scans taken at 2 and 4 hr provide an excellent screening test for detecting fast or slow gastric emptying; the accuracy of 2-hr data was optimal for accelerated emptying and that of the 4-hr data greater for delayed emptying (16).

Krishnaiah et al. sodium chloride core tablets containing either Tc-99m DTPA or Tc-99m SC prepared and compression coated with two different quantities of guar gum. They concluded that Tc-99m DTPA in the evaluation of colonic drug delivery systems containing water-soluble drugs by gamma scintigraphy (17).

Klingensmith et al. evaluated the intrapatient correlation for gastric emptying times with instant oatmeal and scrambled egg meals. The significant correlation between the instant oatmeal and scrambled egg gastric emptying times suggests that similar information is provided by the 2 meals (12).

The medical literature states that solid gastric emptying studies are more sensitive for the detection of gastroparesis than are liquid studies; thus, liquid studies are rarely required (8,18). However, Ziessman et al. investigated in order to determine whether a study of clear liquid gastric empting has added value for the diagnosis of gastroparesis over a study of solid emptying alone.

A total of 101 patients underwent both solid and liquid gastric-emptying studies, acquired sequentially on the same day. They used 300 mL of water with 7.4 MBq [0.2 mCi] of 111 In-diethylenetriaminepentaacetic acid for a 30-min (1-min frames) liquid study followed by a standardized 4-h solid-meal study with a 99mTcsulfur colloid-labeled egg-substitute sandwich meal. They claimed that for the detection of gastroparesis, a 30-min study of clear liquid gastric-emptying has considerable added diagnostic value over a study of solid emptying alone (8). Knight reported radiation exposure as effective dose equivalent (EDE), which is a whole-body weighted average of doses delivered to various body organs and tissue for radiolabeled meals. Radiation dosimetry for nonabsorbable solid labeled with Tc-99m (18.5-37 MBq), Upper large intestine organ radiation dose and effective dose were founded as 0.11 Gy/MBq, 0.024 mSv/MBq respectively (9). 

Gastric emptying scintigraphy still remains the most widely used GI scintigraphic test and has been established as the gold standard for measuring gastric emptying (10,15). Scintigraphy studies are well tolerated and easy to perform. The measurement of gastrointestinal functions by the use of scintigraphy is established in clinical practice and research. The most commonly used test is the gastric-emptying test, which is acknowledged as the gold standard to measure gastric motility and is conducted according to a consensus statement from the national nuclear medicine and motility societies (19,20,21).

## CONCLUSION

Gastric emptying with a solid test meal is the preferred method to assess gastric emptying in older children and adolescents. It is important that the radioactive label remains firmly attached to solid phase. Results show that Tc-99m labeled particulate radiopharmaceuticals binds better to egg compared with other radiopharmaceuticals. Our experiments demonstrated that in addition to Tc-99m sulfur colloid or nanocolloid Tc-99m MAA, and Tc-99m tin colloid also appear to make stable complexes with eggs in acidic environment. It was seen that low binding efficiency of the Tc-99m pertechnetate and Tc-99m DTPA with eggs are not convenient for solid phase GES. Even if Tc-99m DTPA is used different forms or for liquid GES, particulate radiopharmaceuticals are convenient for solid GES.

The best binding efficiency with eggs was found Tc-99m MAA among the particulate and nonparticulate radiopharmaceuticals. This may be related to MAA binding to egg albumin proteins firmly as it becomes denatured during cooking. Tc-99m MAA and other particulate radiopharmaceuticals may be used for gastric emptying studies which could be more practical in routine conditions.

## Figures and Tables

**Table 1:**
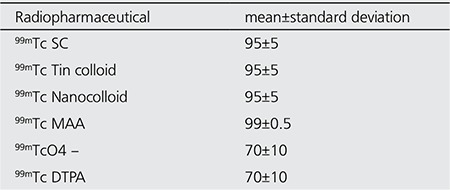
Labeling efficiency at 90 minutes
